# Enhancing Stability and Emissions in Metal Halide Perovskite Nanocrystals Through Mn^2^⁺ Doping

**DOI:** 10.3390/nano15110847

**Published:** 2025-06-01

**Authors:** Thi Thu Trinh Phan, Thi Thuy Kieu Nguyen, Trung Kien Mac, Minh Tuan Trinh

**Affiliations:** 1 Chemistry and Biochemistry Department, Utah State University, 300 Old Main Hill, Logan, UT 84322, USA; phan.trinh@usu.edu (T.T.T.P.); kieu.nguyen@usu.edu (T.T.K.N.); 2 Physics Department, Utah State University, 300 Old Main Hill, Logan, UT 84322, USA; kien.mac@usu.edu

**Keywords:** metal halide perovskite nanocrystals, quantum dots, Mn-doped methylammonium lead bromide, crystal phase transition, PLQY enhancement, perovskite stability, nanocrystal superlattices

## Abstract

Metal halide perovskite (MHP) nanocrystals (NCs) offer great potential for high-efficiency optoelectronic devices; however, they suffer from structural softness and chemical instability. Doping MHP NCs can overcome this issue. In this work, we synthesize Mn-doped methylammonium lead bromide (MAPbBr_3_) NCs using the ligand-assisted reprecipitation method and investigate their structural and optical stability. X-ray diffraction confirms Mn^2^⁺ substitution at Pb^2^⁺ sites and lattice contraction. Photoluminescence (PL) measurements show a blue shift, significant PL quantum yield enhancement, reaching 72% at 17% Mn^2^⁺ doping, and a 34% increase compared to undoped samples, attributed to effective defect passivation and reduced non-radiative recombination, supported by time-resolved PL data. Mn^2^⁺ doping also improves long-term stability under ambient conditions. Low-temperature PL reveals the crystal-phase transitions of perovskite NCs and Mn-doped NCs to be somewhat different than those of pure MAPbBr_3_. Mn^2^⁺ incorporation into perovskite promotes self-assembly into superlattices with larger crystal sizes, better structural order, and stronger inter-NC coupling. These results demonstrate that Mn^2^⁺ doping enhances both optical performance and structural robustness, advancing the potential of MAPbBr_3_ NCs for stable optoelectronic applications.

## 1. Introduction

Metal halide perovskites (MHP) nanocrystals (NCs) or quantum dots are gaining significant interest due to their intriguing optical and physical properties, including bandgap tunability via tuning NC size and chemical compositions, high radiative recombination rate, defect tolerance, high absorption coefficient, high photoluminescence (PL) quantum yield, long-range coherent coupling, and strong Coulomb interaction [1,2,3,4]. These excellent properties offer MHP NCs as building blocks for developing highly efficient optoelectronic devices, for example, light-emitting diodes [5,6], photodetectors [7], photovoltaic cells [8,9,10], lasers [11,12], and single-photon sources [4,13,14]. Three-dimensional MHPs generally have a structure of ABX_3_, where A is a cation and can be Cs^+^ or methylammonium CH_3_NH_3_^+^ (MA), B is a divalent metal ion like Pb^2+^ or Sn^2+^, and X is a halide anion (X = I, Br, Cl). At room temperature, the crystal adopts a cubic phase, consisting of a cubic array of corner-sharing BX_6_ octahedra, with the A-site cation located within the cuboctahedral cavities. High synthesis yields can be achieved using established methods such as hot injection or ligand-assisted reprecipitation (LARP) [15,16]. The most common perovskite NCs are CsPbX_3_ and MAPbX_3_, because of their ease of synthesis. However, MHP structures are soft and chemically unstable; with dynamic and weak surface–ligand binding and susceptibility to anion exchange reactions, their stability has always been a large concern and challenge. MHP NCs are affected by external factors such as light, humidity, ambient temperature, and proton exchange reactions, leading to a decrease in the photoluminescence quantum yield (PLQY) over time, hindering their practical applications. Several strategies have been proposed and implemented to improve the optoelectronic performance of MHP NCs_,_ such as surface passivation, heterostructure formation, A-site doping, B-site doping, and core/shell structures [17,18,19,20]. B-site doping is an effective strategy for enhancing the PLQY of MHP NCs, offering a straightforward synthesis process that preserves the integrity of the perovskite structure. In addition, B-site doping also enables the emergence of new physical phenomena and allows for precise tuning of the optical and electronic properties of the material [20,21,22,23,24,25,26]. Wang et al. doped Eu^3+^ into CsPbCl_3_, and the positions of typical emission bands were tuned by adjusting the Eu^3+^ ion doping amount [27]. In general, metallic ions such as Co^2+^, Cu^2+^, Mn^2+^, Fe^2+^, and rare earth ions are suitable for B-site dopants into MHP NCs; enhancing their optoelectronic properties hold promise for their applications in highly efficient optoelectronic devices [28,29,30,31,32]. Among them, Mn^2+^ is the most sought after, due to its unique chemical and physical properties, such as exhibiting bright and stable PL, introducing magnetic and spintronic properties, and enhancing the optical stability of perovskite materials [21,22,33,34,35,36]. Not all metallic ions can be doped into perovskites. With the structure ABX_3_, the Goldschmidt tolerance factor, $t=\frac{r_{A}+r_{X}}{\sqrt{2}\times (r_{B}+r_{X})}$, should be in the range of 0.76–1.13 to ensure the three-dimensional perovskite structure, where *r_A_*, *r_B_*, and *r_X_* are the radii of atoms A, B, and X, respectively [37,38]. The doped metal species are also constrained by the octahedral coefficient *μ*, defined by $\mu =r_{B}/r_{X}$; the stability range for *μ* is between 0.44 and 0.89 [39,40]. The incorporation of Mn^2+^ into APbX_3_ perovskite crystal lattice at an optimal concentration would increase the tolerance factor, improve the structural stability, and enhance the radiation pathways [37,38]. Liu et al. elucidated the role of the relative strengths of the Mn-Cl bond in the precursor and host lattice in incorporating Mn^2+^ ions into perovskite CsPbX_3_ NCs. They successfully doped Mn^2+^ into CsPbCl_3_ NCs and observed a dual-band emission, which originated from the energy transfer between the Mn^2^⁺ ion and the host [41]. In addition, cation dopants can suppress non-radiative (NR) recombination due to effectively passivating surface and structural defects, leading to an increase in the PLQY of perovskite NCs [35,42]. The mechanism of Mn^2+^ doping is explained through the dynamic exchange of cations and halogen anions. The homologous bond dissociation between Mn-X (halogen) and Pb-X is extremely important, and enables the successful substitution of Pb^2+^ by Mn^2+^ [43,44].

Most Mn^2+^-doped MHP NCs, particularly CsPbBr_3_ and CsPbI_3_, have been synthesized using a hot injection method. While this approach yields high-quality NCs, it requires a high reaction temperature and the precise control of temperature and reaction time and poses significant challenges for scale-up. In this study, we synthesized Mn^2+^-doped MAPbBr_3_ NCs using a simple method, ligand-assisted reprecipitation, at room temperature. We observed PLQY improvement in MAPbBr_3_ NCs with Mn-dopant, reaching an optimal value of 72% at 17% Mn^2+^, an increase of 34% compared to the undoped sample. Mn^2+^ doping can passivate the defects of NCs, replacing the vacancy of cation sites without changing the structure of the perovskite material, resulting in significantly improved optical stability. In addition, to investigate the effect of Mn^2+^ on the optical and structural stabilities, we monitor the long-term stability of the sample under ambient conditions, assess optical properties at low temperatures, and grow NC superlattices from non-doped and Mn-doped MAPbBr_3_ NCs. The improvement in NC quality when using Mn^2+^ doping is a promising strategy for growing superlattices of MAPbBr_3_ NCs via a self-assembly process.

## 2. Materials and Methods

### 2.1. Materials

Lead (II) bromide (99%), methylamine bromide (CH_3_NH_2_Br), manganate (II) bromide (99%), n-octylamine (≥99%), oleic acid (≥90%), N,N-dimethylformamide (DMF), and toluene, didodecyldimethylammonium bromide (DDAB, 98%) were purchased from Sigma-Aldrich (Merck KGaA, Darmstadt, Germany).

### 2.2. Synthesis of Mn-Doped MAPbBr_3_ NCs

The method we used to synthesize xMn-CH_3_NH_3_PbBr_3_ NCs was ligand-assisted reprecipitation (LARP), where x is the mole percentage in the precursors. First, 0.2 mmol of CH_3_NH_3_Br, 0.2(1−x) mmol of PbBr_2_, and 0.2x mmol of MnBr_2_ (x = 0, 0.05, 0.1, 0.15, 0.17, 0.2, and 0.25) were mixed in 5 mL of DMF with 40 μL of oleylamine (OLA) and 400 μL of oleic acid (OA) to generate a precursor solution for the standard manufacturing of MAPbBr_3_ NCs. After that, at room temperature, the precursor solution was injected into toluene at a 1:10 volume ratio. The reaction was left for 10 min to form colloidal MAPbBr_3_ NCs. The product was then centrifuged at 7000 rpm for 10 min. The large crystal aggregated at the bottom was removed. The upper fraction was then further centrifuged at 20,000 rpm for 2 h to discard the supernatant. The bottom containing NCs was then diluted in toluene to make a NC solution. Since the exact amount of Mn^2^⁺ incorporated into the perovskite lattice is unknown, we name the Mn-doped samples as xMn-MAPbBr_3_, where x is the molar ratio of Mn to the total (Pb+Mn).

### 2.3. Surface Passivation

A mixture of 9.2 mg of dimethyldidodecylammonium bromide (DDAB) in toluene (2 mL) was prepared to have a solution of DDAB in toluene (0.01 M). Then, 200 μL of the purified xMn-MAPbBr_3_ NCs and 10 μL of DDAB solution (0.1 M) were mixed and stirred for 1 h at room temperature [45]. We discarded the supernatant and dispersed the precipitate in 5 mL of toluene.

### 2.4. Preparation of NC Superlattice

xMn-MAPbBr_3_ (x = 0 and x = 0.17) NC solutions were used to form a superlattice via the self-assembly method through the gradual evaporation of the solvent. The square pieces of silicon substrate <P-111> 1 cm × 1 cm were placed in a glass Petri dish of a diameter of 90 mm. Approximately 50 μL of the NC solution was dropped onto the Si substrate. To protect it from ambient light and air currents, the Petri dish was covered with a glass lid and loosely wrapped in foil. The superlattice was formed in about 8 to 10 h [45].

### 2.5. Characterizations

X-ray diffractions (XRD) were recorded using an X-ray diffractometer (Rigaku Corp. Tokyo, Japan, Cu Kα with λ = 1.54056 Å, 40 kV, and 40 mA). The samples were prepared by drop-casting NC solution onto a silicon substrate, <P-111>, for measurements.

Atomic force microscopy (AFM) imaging was performed to measure the thickness and morphology of MAPbBr_3_ superlattice, using a silicon tip operating in semi-contact mode from NTEGRA Spectra II (NT-MDT Inc., Moscow, Russia).

Photoluminescence (PL) confocal scanning: The measurement device was equipped with a confocal laser microscope. The excitation and collection were carried out with a 100x-long working distance objective with a numerical aperture of 0.67. The sample was excited via the femtosecond laser pulses at a wavelength of 400 nm or 800 nm, with a pulse duration of <100 fs and a repetition rate of 80 MHz (Mai Tai, Spectra Physics, Newport Corporation, Irvine, CA, USA). The PL image was captured using a Galvo mirror scanning system (Thorlabs, Newton, NJ, USA), and the PL signal was recorded using an EM-CCD camera.

Optical Spectroscopy: The absorption spectra were measured with a UV-VIS spectrophotometer (Shimadzu RF-5301PC, Kyoto, Japan). A femtosecond laser pulse at 400 nm was used to excite the sample for PL spectral measurements. Using a 10× magnification objective for excitation and collection through a long-pass chromatic mirror and color filter, an Ocean Optics spectrometer (Ocean Insight, Orlando, FL, USA) was also used for this measurement. Time-resolved photoluminescent (TRPL) dynamics were obtained using time-correlated single photon counting (TCSPC), PicoHarp300 (PicoQuant, Berlin, Germany). The samples for PL and TRPL measurements were prepared by drop-casting NC solution onto a silicon substrate.

## 3. Results and Discussion

### 3.1. Structural Characterization of Mn-Doped MAPbBr_3_ NCs

**Figure 1:**
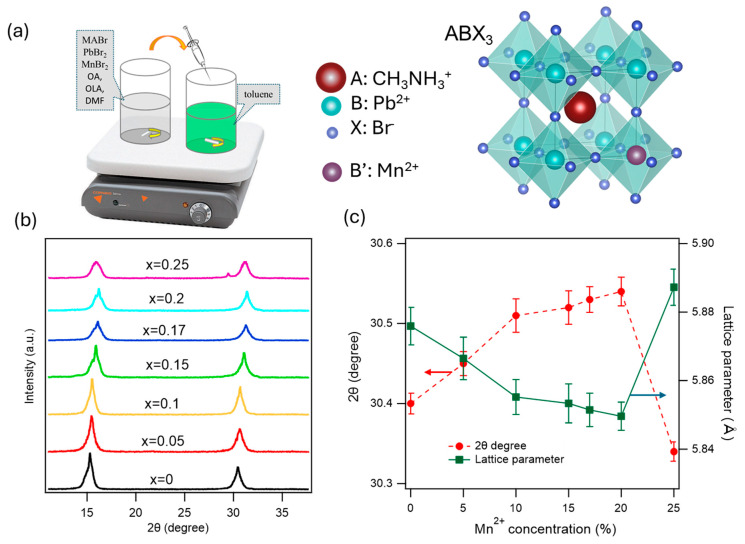
(**a**) Schematic of Mn-doped MAPbBr_3_ NC synthesis and the perovskite crystal structure; (**b**) XRD patterns of xMn-MAPbBr_3_ NCs with different Mn^2+^ concentrations; and (**c**) 2θ degree (red) at (200) plane and calculated lattice parameter (blue) as a function of Mn^2+^ concentration.

The one-pot synthesis of Mn-doped MAPbBr_3_ perovskite NCs using ligand-assisted reprecipitation (LARP) is described in Section 2.2. Briefly, a mixture of precursors including MABr, PbBr_2_, MnBr_2_ (x molar fraction), OLA, and OA with the desired stoichiometric molar concentrations in a polar DMF solvent was injected into a non-polar anti-solvent toluene container under vigorous stirring at room temperature. The crystal growth is due to a reduction in solubility via the anti-solvent, leading to supersaturation and fast nucleation (Figure 1a). The ligands (OA and OLA) prevent uncontrolled aggregation, provide colloidal stability, and passivate the crystal surface. This synthesis method is simple, a room temperature process, and scalable. The NC size can be controlled via the reaction temperature (see Figure S2 Supporting Information). In the Mn-doped perovskite, Mn^2+^ substitutes for the Pb^2+^ cation site. The perovskite structure is maintained even though the cell size is decreased because of the smaller atomic size of Mn^2^⁺ compared to Pb. The shrinkage of the crystal lattice is reflected in a shift in the XRD pattern. The Mn^2+^ ions directly participate in the nucleation process to form MAPb_1−y_Mn_y_Br_3_ NCs, within the limit of the Mn^2+^ concentration, allowing the perovskite structure to be stable. This can be confirmed through the calculation of the tolerance factor and octahedral factor of the perovskite [37,38,46]. These considerations reflect a trade-off between the colloidal stability and the successful incorporation of Mn^2+^ ions.

Figure 1 presents the XRD patterns of xMn-MAPbBr_3_ NCs for various Mn^2+^ concentrations. There are two strong diffraction peaks at around 15.2° and 30.4°, corresponding to the (100) and (200) for both pure and Mn^2+^-doped MAPbBr_3_, confirming the cubic phase of perovskite crystal at room temperature [47,48] (see the similar XRD pattern of the MAPbBr_3_ single crystal and NCs in the Supporting Information, Figure S1). No secondary peaks were observed on the XRD patterns, and the shift in the XRD peaks confirms the effective incorporation of Mn^2+^ into the MAPbBr_3_ lattice. The XRD peak shift increases with the increasing doping concentration up to 20% of Mn^2^⁺, which indicates the incorporation of Mn^2+^ into the crystal lattice. It has been reported that doping can induce structural evolution, especially when the size mismatch between the dopant atom and the substituted atom is large, but the small distortion here shows that the lattice contraction and octahedral tilt are not strong enough to produce a discernible structural change [49]. The XRD peak shift to a larger angle is shown in Figure 1c, with an increasing dopant concentration because of the contraction of the lattice due to the replacement of higher crystal radii Pb^2+^ ions (1.12–1.19 Å) by smaller Mn^2+^ ions (0.8 Å) [50,51,52]. However, at x = 0.25, the XRD behavior is different. It shifts to a lower angle and a small new peak appears at 28.5°. The different behavior of the XRD shift at x = 0.25 is unclear; it could be at a high Mn^2^⁺ concentration the Mn^2+^ ions do not substitute Pb^2+^ ions, but rather form segregated crystal phases of MAPbBr_3_ and MAMnBr_3_, which will lead to a lower PLQY. A higher Mn^2+^ concentration pushes the tolerance factor and octahedral coefficient, which are in the range of 0.76–1.13 and 0.44–0.89, respectively [36,37], out of the stable range. The calculated tolerance factor and octahedral coefficient for MAPbBr_3_ and MAMnBr_3_ are given in the Supporting Information. Further research is needed to reveal the crystal structures at high Mn^2^⁺ concentrations.

At room temperature, the MAPbBr_3_ crystal has the cubic phase, and the lattice parameter *a* is calculated from the XRD peak (200) using the formula [53]: $a=d.\;\sqrt{h^{2}+k^{2}+l^{2}}$, where (*h k l*) are the Miller indices and d is the interplanar spacing, which is calculated using Bragg’s law *nλ =* 2*d.sinθ*. In the XRD, *θ* is the diffraction angle and λ is the wavelength of the incident X-ray (1.54 Å for Cu Kα) for the first-order diffraction n = 1. In Figure 1c, the calculated lattice parameter decreases with increasing Mn^2^⁺ concentrations, except for the 25% Mn^2^⁺ dopant. This indicates that the smaller dopant Mn^2+^ ions are substituting for Pb^2+^ ions, causing the contraction of the lattice. This observation is consistent with Vegard’s law, which states that the lattice parameter changes linearly with composition of a solid [54].

### 3.2. Optical Characterization of Mn-Doped MAPbBr_3_ NCs

**Figure 2:**
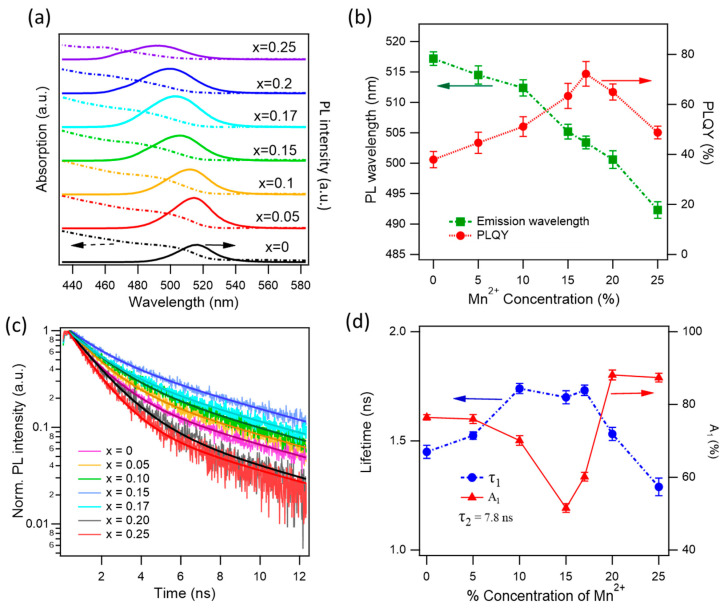
Optical characterizations of xMn-MAPbBr_3_ perovskite NCs. (**a**) Absorption (dashed) and emission (solid) spectra with the different Mn^2+^ doping concentrations. The PL spectra were measured at 400 nm excitation. (**b**) Mn^2+^ concentration dependence of the emission wavelength (green) and PLQY (red). (**c**) Normalized PL dynamics at 400 nm excitation, excitation pulse 100 fs. (**d**) Non-radiative lifetime (τ_1_) and its amplitude percentages obtained from global fittings, the radiative lifetime was held the same for all fits. Fitted radiative lifetime returns a value of 7.8 ± 1 ns.

Figure 2 presents the optical characterizations of xMn-MAPbBr_3_, with the Mn^2+^ concentration increasing from x = 0 to 0.25. We observe a gradual blue shift in the emission wavelength from 517 to 492 nm, in agreement with the previous observations [55]. The absorption, emission, and PLQY measurements were carried out on NCs in toluene solutions. The incorporation of Mn^2+^ ions in the crystal lattice may also perturb the electronic band structure of the perovskite crystal causing spectral shift, although it does not introduce new energy levels. While Mn-dopant in CsPbCl_3_ introduced new energy levels and dual-band emissions arose [21,56], the absence of Mn^2+^ emissions in Mn-doped CsPbBr_3_ or MAPbBr_3_ was due to the negligible difference between the ^4^T_1_ and ^6^A_1_ band gap of the d-state of Mn^2+^ ions and the transition band gap of pure APbBr_3_ crystals [57,58,59].

As the Mn^2+^ doping concentration increases, the PLQY increases from 38% for the pure MAPbBr_3_ to 72% for the 17% Mn^2+^ concentration. Further increasing the Mn^2+^ doping concentration results in a drop in the PLQY. At 25% of Mn^2+^, the PLQY is 49% (Figure 2b). The significant PLQY enhancement with increasing Mn^2+^ concentration to MAPbBr_3_ NCs indicates the efficient reduction in NR recombination pathways. It has been suggested that Mn^2+^ ions fill the Pb^2+^ vacancies and passivate the dangling bonds and defects of the crystal surface [55]. Further increasing the Mn^2+^ concentration above 20% leads to a decrease in the PLQY, possibly due to the strong distortion of the crystal lattice introducing new defects, or may result in the segregation phase of MAPbBr_3_ and MAMnBr_3_, forming domain boundaries and defects as the reverse change in the XRD shift (Figure 1c).

To have better insight into the reduction in NR recombination pathways induced by Mn^2+^ doping, we measure the time-resolved PL (TRPL) dynamics at various Mn^2+^ concentrations, as shown in Figure 2c. The measurements were performed on thin films, and the resulting dynamics reveal two decay time constants corresponding to radiative and NR recombination. For simplicity, we assume that the radiative rate remains constant across different Mn^2+^ concentrations. We performed global fitting of the dynamics, keeping the radiative lifetime fixed for all traces. The fit is used a biexponential function,

(1) $$I\left(t\right)=A_{1}\exp\left(-\frac{t}{\tau_{1}}\right)+A_{2}\exp\left(-\frac{t}{\tau_{2}}\right),$$

where (*A*_1_, $\tau_{1}$) and (*A*_2_, $\tau_{2}$) are the amplitudes and lifetimes of NR and radiative decay channels, respectively. The obtained fitting constants are displayed in Figure 2d, and the radiative lifetime is 7.8 ± 0.1 ns for all traces. The NR lifetime increases from 1.4 to 1.7 ns (at x = 0.1, 0.15, and 0.17), further increasing the Mn^2^⁺ concentration results in the reduction in the NR lifetime. The radiative lifetime of excitons in perovskite NCs has been reported to vary largely from a few to tens of ns, depending on the NC size and excitation conditions. The NR lifetime was reported to be ~1.5 ns, in agreement with our observation [60,61]. The most important observation here is the percentage of the NR amplitude constant, *A*_1_. This represents the weight of the NR channel. As can be seen, the weight of NR recombination is 76% for non-doped NCs and drops to 51% at x = 0.15, and rises to ~87% at x = 0.2 and 0.25. The lowest NR component at x = 0.15 is consistent with the maximum PLQY at x = 0.17. It is worth noting that while the PLQY measurements were performed on solution samples, the TRPL dynamics were measured on NC films, which may lead to some discrepancies between the two measurements. Mn^2^⁺ doping, at an optimal concentration, effectively reduces non-radiative recombination by filling Pb^2^⁺ vacancies and passivating defects and dangling bonds. Perovskite materials are intrinsically soft and ionic, which makes them prone to ion migration and phase segregation. Since Mn^2^⁺ has a smaller ionic radius than Pb^2^⁺, it can substitute for Pb in the lattice, leading to a more compact and rigid crystal structure that enhances overall stability. Moreover, Mn doping helps to suppress halide ion migration by anchoring halide ions or introducing lattice distortions that act as barriers to ion movement. It also passivates halide vacancies and reduces deep-level defects, thereby improving optical quality and enhancing the long-term durability of the perovskite structure [62,63].

### 3.3. Optical Stability of Mn-Doped MAPbBr_3_ NCs

**Figure 3:**
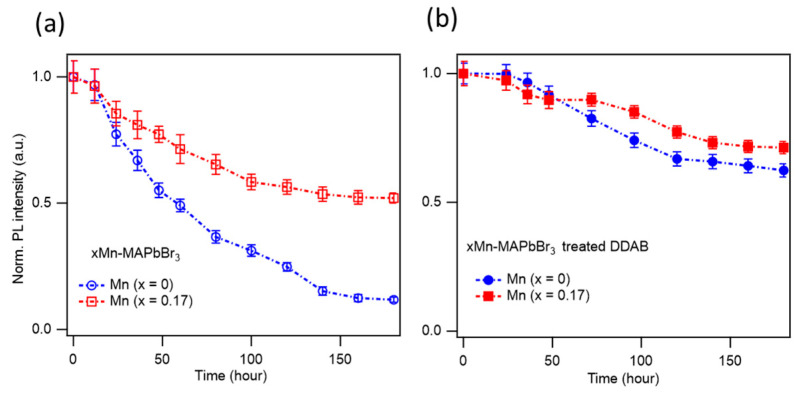
Optical stability of xMn-MAPbBr_3_ samples with x = 0 and x = 0.17 over 180 h for non-treated NCs (**a**) and DDAB-treated NCs (**b**). The samples were diluted in toluene solvent and drop-cast on Si substrate for storage and measurement in ambient conditions.

As discussed above, Mn^2^⁺ doping in MAPbBr_3_ NCs enhances the PLQY by reducing non-radiative decay channels. Mn^2^⁺ doping also improves the long-term optical stability. Figure 3 shows the PL stability of MAPbBr_3_ NC films stored under ambient conditions over 180 h. The as-synthesized xMn-MAPbBr_3_ NCs exhibit PL intensity degradation, with the PL intensity retaining only 12% and 52% of the initial value for x = 0 and x = 0.17, respectively. This result indicates that Mn-doped perovskite NCs are significantly more stable than their undoped counterparts. To further enhance the optical stability, we treated the NCs with DDAB. After 180 h under ambient conditions, the PL intensities of the DDAB-treated NCs with x = 0 and x = 0.17 retained 62% and 72% of their original values, respectively, as shown in Figure 3b. A previous study showed that DDAB surface passivation effectively reduces the surface and defect states, enhances optical stability against light-induced PL quenching, and improves long-term stability [64]. The DDA⁺ cations can replace the native oleic acid and oleylamine ligands on the NC surface or fill in for missing ligands, thereby protecting the NCs from environmental factors such as humidity, oxidation, and other external degradation sources. Therefore, Mn^2^⁺ doping improves the PLQY and the optical stability of MAPbBr_3_ NCs by filling Pb^2^⁺ vacancies, passivating defects, reducing ion migration due to the stronger Mn–X bonds compared to Pb–X bonds, and enhancing the overall crystal robustness by minimizing lattice distortion.

### 3.4. Optical Properties at Low Temperature and Crystal Phase Transitions

**Figure 4:**
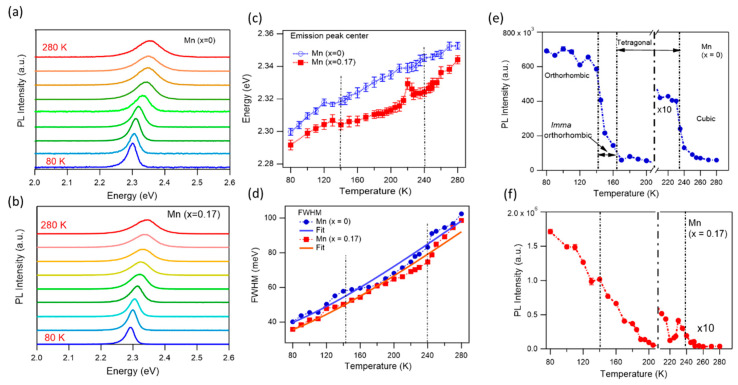
Temperature dependence of optical properties from 80 to 280 K. PL spectra of non-doped (**a**) and 17% Mn-doped (**b**) MAPbBr_3_ NCs. (**c**) PL emission peak maxima in energy; (**d**) emission line widths (FWHM) with the fits from exciton–phonon coupling equation; and (**e**,**f**) PL intensities for non-doped (blue) and 17% Mn-doped (red) NCs. Vertical dashed lines indicate the temperatures where the crystal phase transition happens. The dot-dashed line at 210 K in (**e**,**f**) represents where the data at temperatures > 210 K were magnified by 10×.

Incorporating Mn^2+^ into MAPbBr_3_ NCs may also affect crystal structure, therefore affecting crystallographic phases and phase transition temperatures. To investigate optical properties at different crystal phases, we measure the PL spectra from 80 K to 280 K for two samples, non-doped and 17% Mn-doped NCs. The latter case exhibits the highest PLQY for Mn-doped samples. Figure 4 presents the PL spectra, PL intensities, wavelengths, and line widths of these two samples at different temperatures. Similarly to previous observations in MHP NCs, the PL intensities decreased, the maximum PL peak blue shifted (emission energy increased), and the spectral line width broadened with increasing temperatures [65,66]. Notably, the observed blue shift in PL with increasing temperature is opposite to the typical behavior seen in conventional semiconductors, where lattice expansion generally leads to a red shift. In conventional semiconductors, the valence band maxima (VBM) and conduction band minima (CBM) arise from bonding and antibonding orbital pairs, respectively. As the temperature increases, thermal expansion causes the crystal lattice to dilate, resulting in a reduction in the bandgap energy, which can be described using the Varshni equation [67]. In contrast, the electronic structure of MAPbBr_3_ crystals is fundamentally different. The VBM originates from a strong antibonding interaction between the Pb 6s and Br 4p orbitals, while the CBM arises from an antibonding combination of the Pb 6p and Br 4s orbitals [40,65,68]. As the lattice expands with temperature, the VBM shifts downward in energy due to its antibonding nature (weakened antibonding interactions between atoms), whereas the CBM is relatively insensitive to lattice deformation. This asymmetry leads to an overall increase in the bandgap with temperature. Both undoped and Mn-doped MAPbBr_3_ NCs exhibit similar temperature-dependent behavior. However, an anomalous behavior is observed in Mn-doped MAPbBr_3_ at around 220 K, where a sharp blue shift is followed by a red shift at 227 K. This irregular trend coincides with a threefold drop in PL intensity in the same temperature range (Figure 4f). This observation suggests a possible phase transition occurring near 220 K. Further investigation is needed to clarify the origin of this unusual bandgap and PL intensity behavior in Mn-doped MAPbBr_3_ NCs in this temperature range.

The PL line width (FWHM) broadening observed with temperature originates from enhanced exciton–phonon interaction, as more phonons participate in the emission process at higher temperatures (Figure 4d). The temperature-dependent line width broadening is nearly identical for both undoped and Mn-doped MAPbBr_3_ NCs (Figure 4d). Although both acoustic and longitudinal optical (LO) photons can contribute to homogeneous PL line width broadening, LO phonons have been reported as the dominant factor in perovskite NCs [66]. To deepen our insight into the role of exciton–phonon coupling, we fit the FWHM as a function of temperature, Γ(T), using the following equation [69,70]:

(2) $$\Gamma \left(T\right)=\Gamma_{0}+\Gamma_{ac}+\Gamma_{LO}=\Gamma_{0}+\gamma_{ac}T+\gamma_{LO}N_{LO}\left(T\right),$$

where $\Gamma_{0}$ is the inhomogeneous PL broadening, which is temperature-independent and arises from the NC size distribution. $\Gamma_{ac}$ and $\Gamma_{LO}$ are homogeneous broadening contributions from acoustic and longitudinal optical phonon scattering, respectively. $\gamma_{ac}$ and $\gamma_{LO}$ are the exciton–phonon coupling strengths for acoustic and LO phonons, respectively. The phonon population follows the Bose–Einstein distribution:

(3) $$N_{LO}\left(T\right)=\frac{1}{e^{\frac{E_{LO}}{K_{B}T}}-1},$$

where $E_{LO}$ is the LO phonon energy and $k_{B}$ is the Boltzmann constant. Using Equation (2) to fit the FWHM in Figure 4d, we obtained the parameters $\Gamma_{0},\;\gamma_{ac}$, $E_{LO}$, and $\gamma_{LO}$ that are 32 ± 5 meV, 0.07 ± 0.02 meV/K, 24 ± 4 meV, and 80 ± 7 meV for non-doped MAPbBr_3_ NCs, and 29 ± 6 meV, 0.06 ± 0.0 meV/K, 25 ± 5 meV, and 85 ± 7 meV for Mn-doped MAPbBr_3_ NCs. These values are consistent with the previous observations in perovskite film and NCs, except the acoustic phonon couplings in our case are much lower in comparison with the report from Ref. [66]. This low acoustic phonon contribution is also consistent with the earlier studies [69,70]. Some discrepancies between the fitted curves and the experimental data in Figure 4d may arise from the asymmetric PL line shape, which could be attributed to a low-energy shoulder peak. This feature likely originates from shallow trap states in the perovskite NCs, as discussed in the Supporting Information, Figure S4.

Figure 4e,f presents the temperature-dependent PL intensities of the two samples. As the temperature increases, the PL intensities decrease due to enhanced NR recombination. At high temperatures, strong carrier–phonon interactions enable electrons or holes to acquire sufficient thermal energy to overcome potential barriers and transition into defect states, facilitating NR recombination. Conversely, at low temperatures, the MAPbBr_3_ crystal structure becomes more ordered as the MA^+^ cations, which rotate freely at room temperature, are immobilized. This structural ordering can lead to higher emission rates and narrower spectral line widths. Moreover, when the temperature changes across the crystallographic phase transitions, the relative alignment between excited carriers and trap energy levels shifts. As a result, we expect abrupt changes in the PL intensity due to variations in NR activation across the phase transition points. It has been reported that bulk MAPbBr_3_ perovskites exhibit three distinct crystallographic phases: below 144 K is the orthorhombic (*Pnma*) phase, between 144 and 237 K is the tetragonal phase (*I4/mcm*), and above 237 K is the cubic phase (*Pm*$\bar{3}$*m*) [71]. However, there has been debate regarding a transient phase within the 148–155 K range, which has been attributed to phase coexistence [72,73]. More recently, Abia et al. employed neutron diffraction to sequentially analyze the crystallographic phase in this temperature range and identified the phase as orthorhombic *Imma* [74]. MAPbBr_3_ perovskites in nanostructured forms, such as NCs, are suggested to suppress noticeable phase transitions, likely due to their reduced crystal size, which inhibits structural transformation. As a result, no clear PL intensity change is typically observed across the known phase transition temperatures [66]. We speculate that the absence of distinct PL intensity changes in NCs across the phase transition points arises from effective surface passivation with a high PLQY, which minimizes the influence of defects or traps across different crystal phases. To see the crystal transition phases, we intended to keep the sample in ambient conditions for one week before measurements, starting from a low PLQY (see Figure 3). The phase transition temperatures for non-doped MAPbBr_3_ NCs are clear (Figure 4e). During the orthorhombic-to-tetragonal phase transition, the PL intensity decreases by nearly 80-fold. Within the *Imma* orthorhombic phase (140–160 K), the PL intensity gradually decreases. At the tetragonal-to-cubic transition (~135 K), the PL intensity drops by about fivefold. Within a single phase, the PL intensity remains relatively stable. In contrast, Mn-doped MAPbBr_3_ NCs do not exhibit a clear orthorhombic-to-tetragonal phase transition, Instead, the PL intensity decreases steadily with increasing temperature. An anomalous change is observed at 220 K, accompanied by a corresponding shift in the PL peak energy (see Figure 4c). A sharp drop in the PL intensity, approximately eightfold, occurs during the transition from the tetragonal to cubic phase. These results demonstrate that crystal phase transitions can be effectively tracked through temperature-dependent changes in the PL intensity.

### 3.5. Superlattice Formation of xMn-MAPbBr_3_ NCs

**Figure 5:**
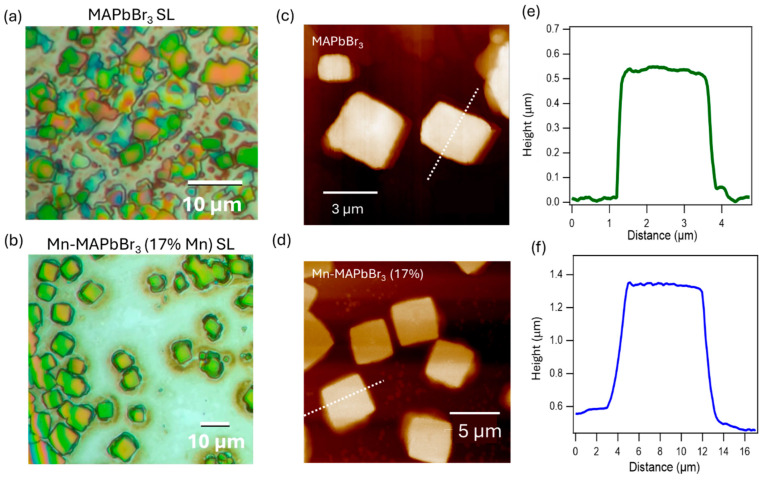
Superlattice characterization for non-doped (top row) and 17% Mn-doped (second row) MAPbBr_3_ NCs. (**a**,**b**) Optical images of superlattices in the best area of SL formation, scale bar: 10 mm. (**c**,**d**) AFM images were obtained using semi-contact mode. (**e**,**f**) AFM cross-sections were obtained via the white dashed lines in (**c**,**d**).

NCs can self-assemble into highly ordered and periodic structures known as superlattices (SLs). This artificial crystal-like arrangement facilitates inter-NC coupling, giving rise to emergent collective electronic, optical, or transport properties that are not present in uncoupled NCs. Most SLs reported to date have been formed using all-inorganic perovskite NCs, such as CsPbBr_3_ synthesized via the hot injection method, which yields high crystal quality and uniformity [75]. The formation of high-quality superlattices relies heavily on the NCs’ size and shape uniformity, as well as their surface chemistry. In contrast, hybrid organic–inorganic perovskites like MAPbBr_3_, typically synthesized using the LARP method, often result in lower crystal quality and hence affect superlattice growth. However, as discussed above, Mn^2^⁺ doping in MAPbBr_3_ has been shown to enhance NC quality, increase quantum yield, and improve material stability, factors that can promote the formation of higher-quality SLs. Furthermore, post-synthesis treatment with DDAB effectively passivates the NC surface, providing favorable conditions for self-assembly. In this study, we compared superlattice formation using pristine MAPbBr_3_ NCs and Mn^2^⁺-doped MAPbBr_3_ NCs (17% Mn^2^⁺), both treated with DDAB. Optical microscopy images (Figure 5) reveal that Mn-doped NCs form well-defined SLs with a regular square shape, larger SL sizes (7–9 µm vs. 2–4 µm for the non-doped NCs), higher uniformity, and clearer backgrounds. Atomic force microscopy (AFM) shows that SLs from pristine NCs have dimensions of ~3 µm in size and ~0.55 µm in thickness, whereas SLs from Mn-doped NCs reach ~8 µm in size and ~0.8 µm in thickness. Additionally, the surface of Mn-doped SLs is noticeably flatter. Additional optical images are given in the Supporting Information, Figures S5 and S6.

**Figure 6:**
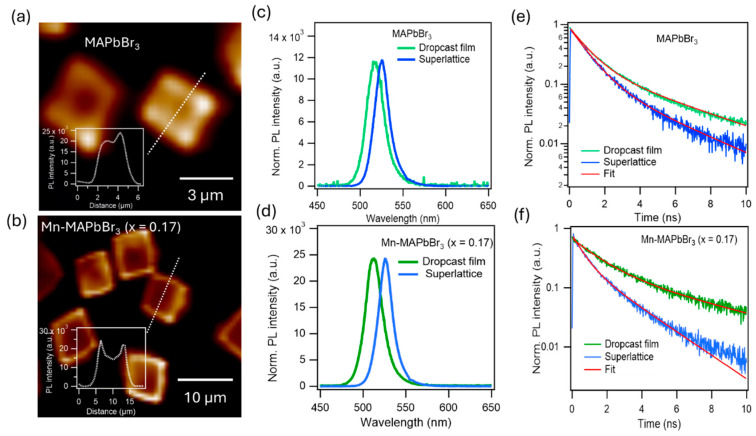
Optical characterization of superlattices grown from non-doped (top row) and 17% Mn-doped (bottom row) MAPbBr_3_ NCs. (**a**,**b**) PL confocal image obtained using two-photon fluorescence (2PF); the excitation laser is at 800 nm. Inset: the profiles of individual SLs indicated by the white dashed lines. (**c**,**d**) Emission spectra of SLs and drop-cast films. (**e**,**f**) PL dynamics of the SLs and drop-cast film NCs.

To have a better PL imaging resolution, we performed two-photon fluorescence (2PF) confocal imaging, using a femtosecond laser with 800 nm excitation. Figure 6a,b present the PL images of SLs grown from non-doped and Mn-doped MAPbBr_3_ NCs. Notably, 2PF intensities are localized higher at the edges of the Mn-doped SLs, which may indicate improved surface passivation at the edge surfaces. To investigate the strong inter-NC coupling, we measured the PL spectra and dynamics of the randomly oriented NC film via drop-casting and compared them to those from SLs; the data are given in Figure 6c,d. For the non-doped NCs, the SL PL spectrum is 8 nm red-shifted compared to that from the drop-cast film. The redshift indicates the strong coupling of NCs in SLs, resulting in the weakening of quantum confinement. For the Mn-doped MAPbBr_3_ NCs, the redshift in SLs is larger, 13 nm, in comparison to that of the drop-cast film. The larger redshift in Mn-doped NC superlattices indicates that the Mn facilitates the stronger inter-NC coupling. The PL dynamics are presented in Figure 6e,f. The double exponential fits to the dynamics returns the lifetime constants of t_1_ and t_2_ of the samples that are given in Table 1. As can be seen, the time constants for SLs are always shorter than those of the random orientation NC film. The observations suggest that the exciton can be delocalized in the SLs, resulting in exciton–exciton annihilation. For the Mn-doped MAPbBr_3_ NCs, the time constant is much shorter in comparison to that of a non-doped sample. Together with the stronger spectral redshift, we can conclude that Mn dopant facilitates the stronger inter-NC coupling.

## 4. Conclusions

In summary, we demonstrate that controlled Mn^2^⁺ doping in MAPbBr_3_ NCs significantly enhances their optical performance and structural stability. Mn^2^⁺ ions are successfully incorporated into the perovskite lattice at concentrations of up to 25%, inducing lattice contraction without compromising the crystal integrity or optical properties. This doping results in a notable increase in the photoluminescence quantum yield, reaching a maximum of 72% at 17% Mn^2+^ doping, and a reduction in non-radiative recombination, as confirmed by PL dynamics and time-resolved measurements. Mn^2^⁺ doping also improves the ambient optical stability of the NCs, which is further enhanced by surface passivation using DDAB. Temperature-dependent PL studies reveal both conventional and anomalous emission behaviors in the Mn-doped samples. Structurally, Mn^2^⁺ doping enhances the robustness of MAPbBr_3_ via filling vacancies and trap states and substituting Pb^2^⁺ sites without disrupting the lattice framework. Moreover, Mn^2^⁺ incorporation promotes the self-assembly of MAPbBr_3_ NCs into superlattice structures, leading to larger nanocrystals and improved crystallinity. These findings offer valuable insights into the role of transition metal doping in tuning the optical and structural properties of perovskite NCs, paving the way for the development of doped perovskites with improved functionality for lighting and optoelectronic applications.

## Figures and Tables

**Table 1:** Time constants for different samples.

	MAPbBr_3_		Mn-Doped MAPbBr_3_ (x = 0.17)	
Time constants	t_1_ (ns)	t_2_ (ns)	t_1_ (ns)	t_2_ (ns)
Drop-cast film	1	4.4	1.46	5.9
Superlattice	0.9	3.4	0.6	2.2

## Data Availability

The original contributions presented in this study are included in the article. Further inquiries can be directed to the corresponding author.

## Supplementary Materials

The following supporting information can be downloaded at: https://www.mdpi.com/article/10.3390/nano15110847/s1. Figure S1. XRD patterns of the MAPbBr3 single crystal (red) and NCs (blue). Figure S2. MAPbBr3 nanocrystals were synthesized with tunable sizes controlled by the reaction temperature. Figure S3. The correlation between the lattice parameter (blue) and the PL shift as a function of Mn concentration. Figure S4. The emission spectra of MAPbBr3 NCs and the fits with a double Gaussian function. Non-doped NC at 80 K (a) and 280 K (b), Mn-doped NCs (x = 0.17) at 80 K (c) and 280 K (d). Figure S5. Optical images of non-doped NC superlattices at different locations. Figure S6. Optical images of Mn-doped (x = 0.17) NC superlattices at different locations.
