# Supplementary material for: Enhancing Stability and Emissions in Metal Halide Perovskite Nanocrystals Through Mn2⁺ Doping
Source: Nanomaterials (Basel). 2025 Jun 1;15(11):847. doi: 10.3390/nano15110847 (PMC12158230; doi:10.3390/nano15110847)
Supplement: Supplementary file 1 [file nanomaterials-15-00847-s001.zip › nanomaterials-3648841-supplementary.pdf]

Supporting Information

Enhancing Stability and Emission in Metal Halide Perovskite Nanocrystals through Mn^{2+} Doping

T. Thu Trinh Phan[1], T. Thuy Kieu Nguyen[1], T. Kien Mac[2], M. Tuan Trinh[1]*

[1]Chemistry and Biochemistry Department, Utah State University, 300 Old Main Hill, Logan, UT, USA.

[2]Physics Department, Utah State University, 300 Old Main Hill, Logan, UT, USA.

1. XRD of $MAPbBr_3$ single crystal and nanocrystals

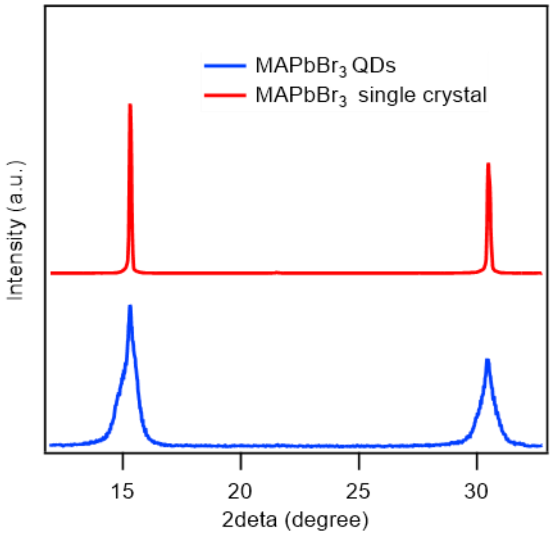

Figure S1. XRD patterns of the $MAPbBr_3$ single crystal (red) and NCs (blue). The similar diffraction peaks indicate the same crystal phase of the single crystal and NCs at room temperature. The peak broadening in NCs is due to the random orientation of NCs in a film.

2. Nanocrystal synthesis at different temperatures.

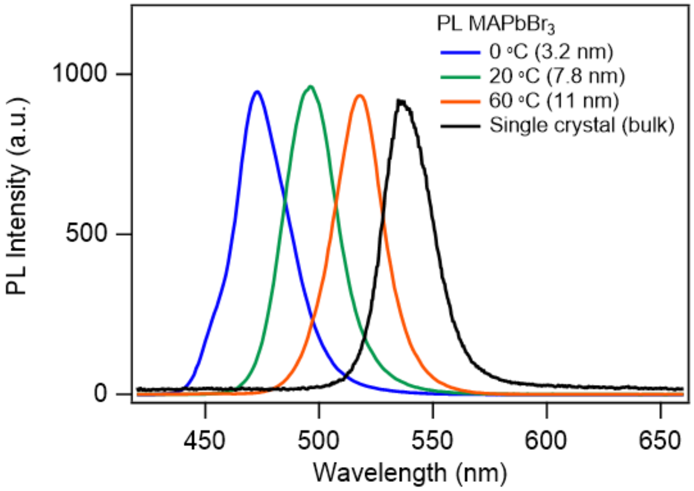

Figure S2. $MAPbBr_3$ nanocrystals were synthesized with tunable sizes controlled by the reaction temperature. The observed blue-shifted emission compared to the bulk material indicates quantum confinement, as the nanocrystal size is smaller than the exciton Bohr radius.

3. Comparison lattice parameter and PL blueshift as a function of Mn concentration

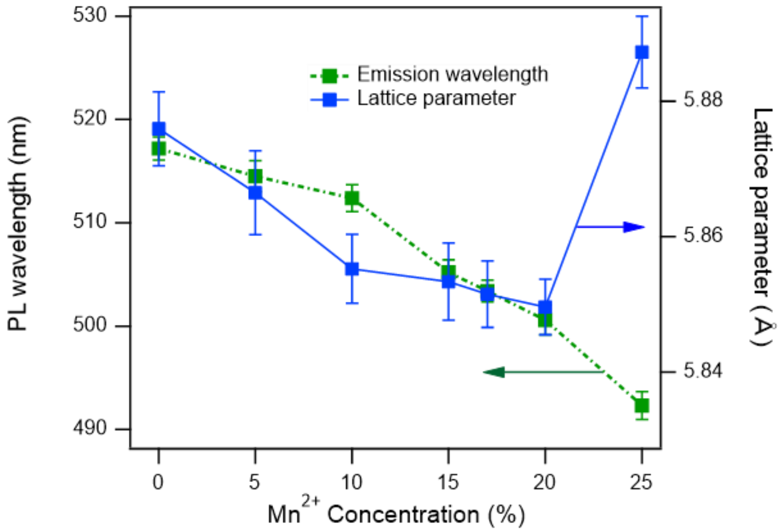

Figure S3. The correlation between the lattice parameter (blue) and the PL shift as a function of Mn concentration. Up to 20% Mn incorporation, the lattice contraction closely correlates with the blueshift in PL emission. It is worth noting that the origin of this blueshift is complex and may arise from the introduction of new electronic states within the $MAPbBr_3$ crystal structure due to Mn doping.

4. Tolerance factor and octahedral coefficient calculations

The structure ABX_3 the Goldschmidt tolerance factor given by:

$$t = \frac{r_A + r_X}{\sqrt{2} * (r_B + r_X)},$$

r_A = ionic radius of A-site cation, MA ≈ 2.17 Å

r_B = ionic radius of B-site cation, Pb ≈ 1.19 Å

r_X = ionic radius of X-site anion, Br ≈ 1.96 Å

for $MAPbBr_3$

t = 0.927

The octahedral coefficient μ, defined by $\mu = r_B/r_X$, the stability range for μ is between 0.44 and 0.89.

$\mu = r_B/r_X = 0.607$

When replacing Pb^{2+} by Mn^{2+} to form $MAMnBr_3$, ionic radius of Mn^{2+} ≈ 0.66 Å.

Tolerance factor, t = 1.11

Octahedral coefficient μ = 0.336.

5. Asymmetric spectral feature analyzing

The emission spectra are asymmetric and can be characterized by two distinct peaks that can be resolved by a double Gaussian function fitting

$$f(E) = A_1 e^{-\left(\frac{E-E_1}{W_1}\right)^2} + A_2 e^{-\left(\frac{E-E_2}{W_2}\right)^2}$$

where E_1 and E_2 are the peak energies of each Gaussian peak, W_1 and W_2 are the Gaussian linewidth of peak 1 and peak 2, respectively.

For non-doped $MAPbBr_3$ NCs, the fit returns the energy peaks are 2.29 and 2.30 eV for the spectrum at 80 K, and 2.26 and 2.35 eV for the spectrum at 280 K. The fit values are given in the figure. The lower energy band emerged at a lower temperature. The origin of the asymmetric spectral feature in perovskite is controversial. It could originate from the crystal structure transitions in these perovskite samples. The lower energy emission band was assigned to a shallow-defect emission, originating from cation and Br vacancy defects. Multiple peaks from the PL spectrum could also come from the fine structure of the perovskite nature in combination with exciton-phonon interaction resulting in asymmetric features.

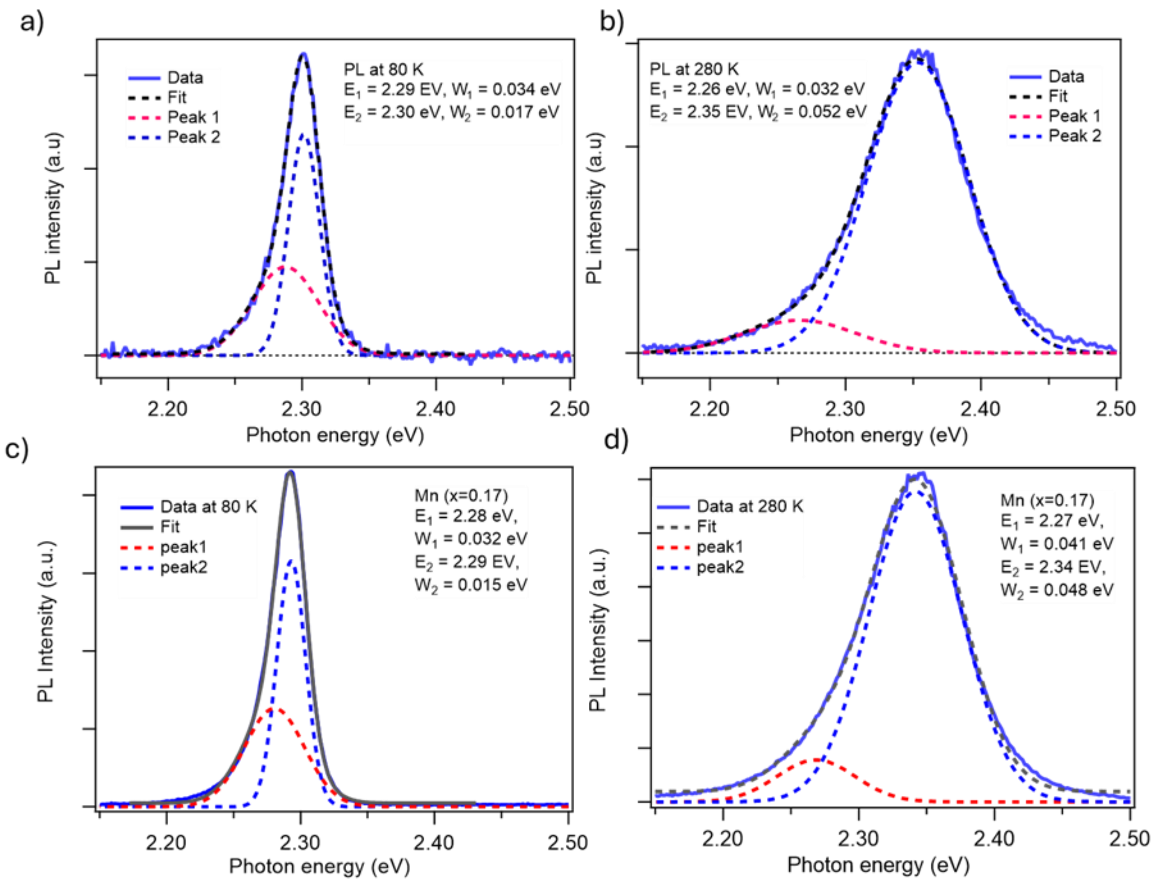

Figure S4. The emission spectra of $MAPbBr_3$ NCs and the fits with a double Gaussian function. Non-doped NC at 80K (a) and 280 K(b), Mn-doped NCs (x = 0.17) at 80 K (c) and 280 K (d).

6. Additional optical images for superlattices

Pristine $MAPbBr_3$ SLs

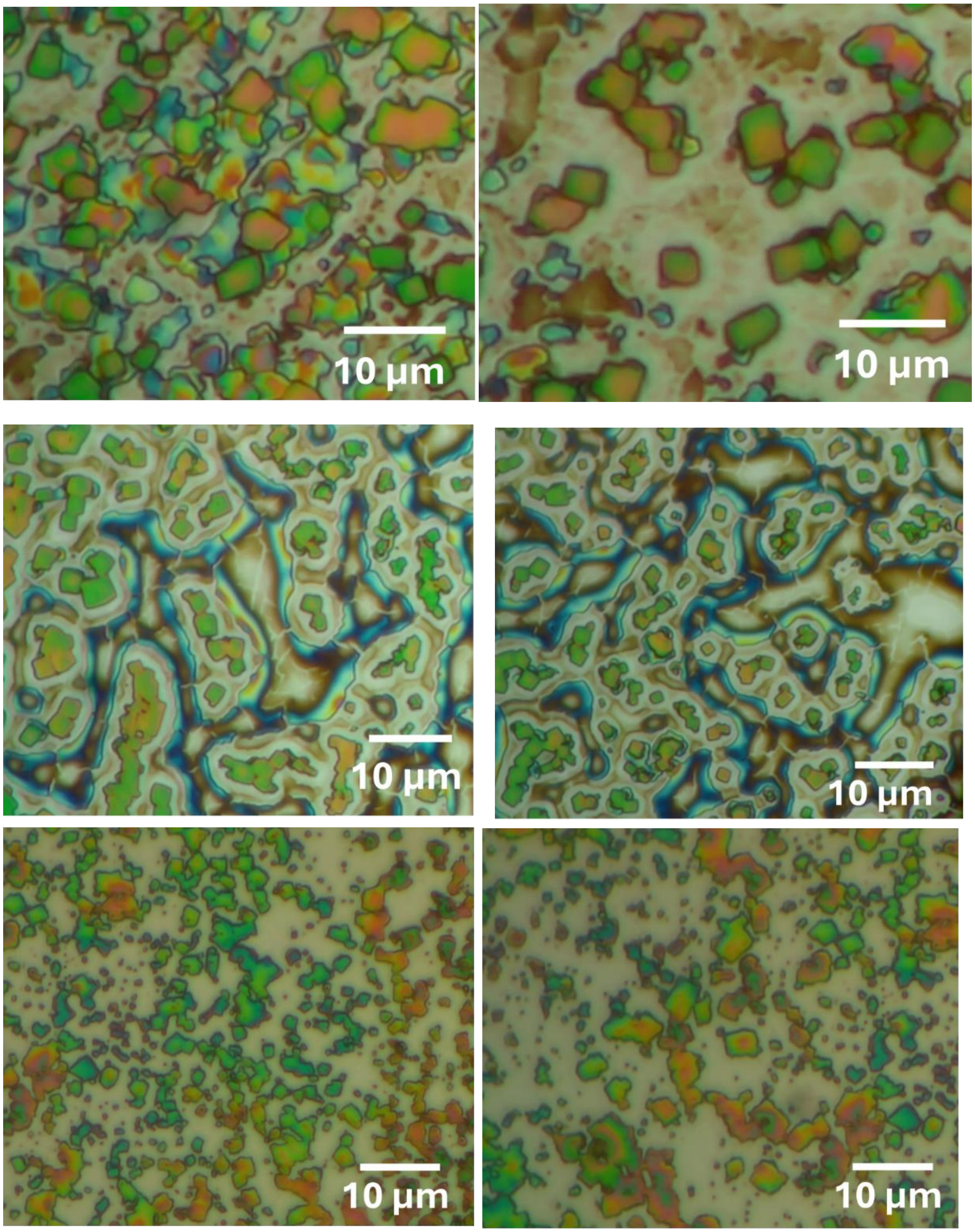

Figure S5. Optical images of non-doped NC superlattices at different locations.

xMn-$MAPbBr_3$ (x=0.17) SLs

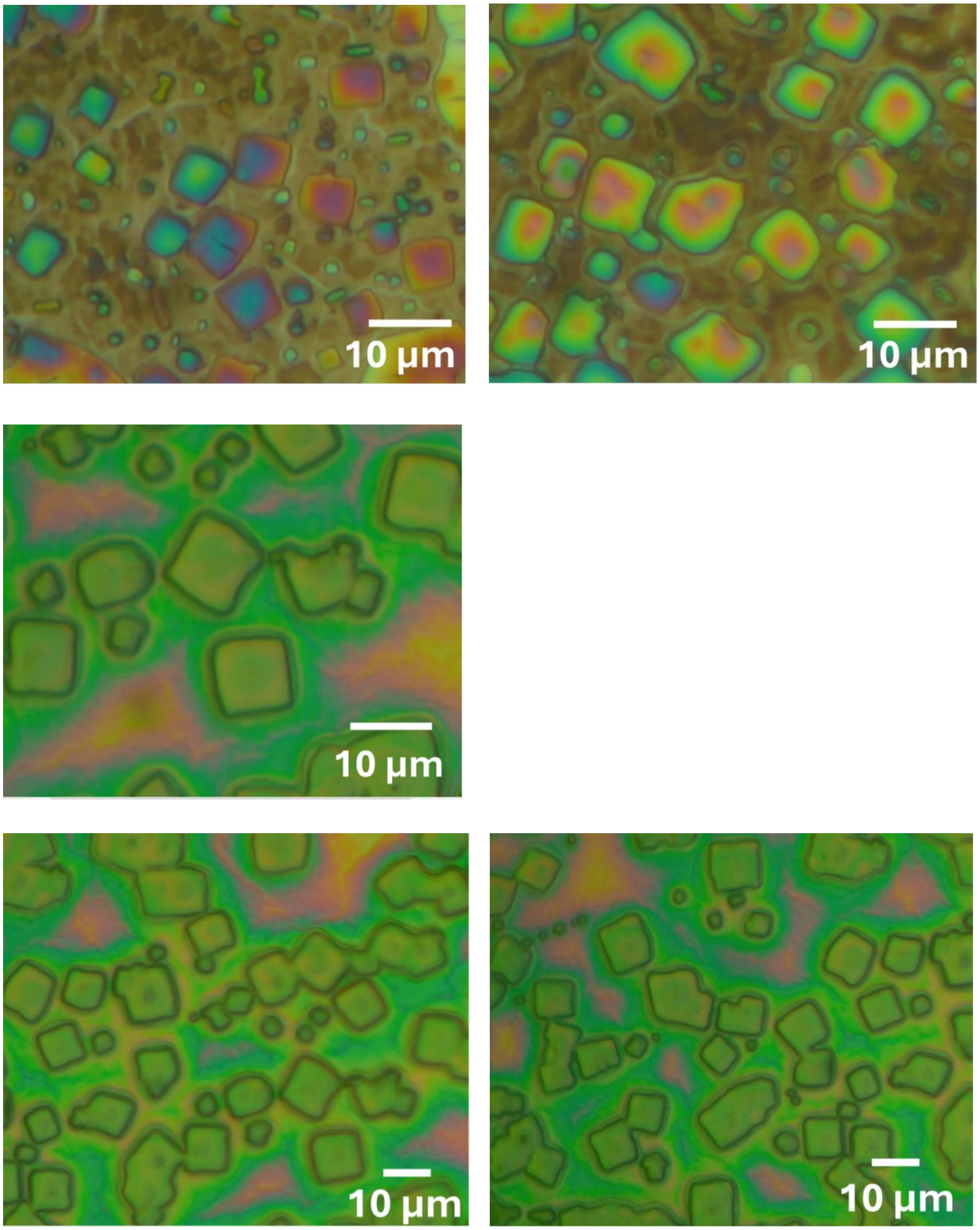

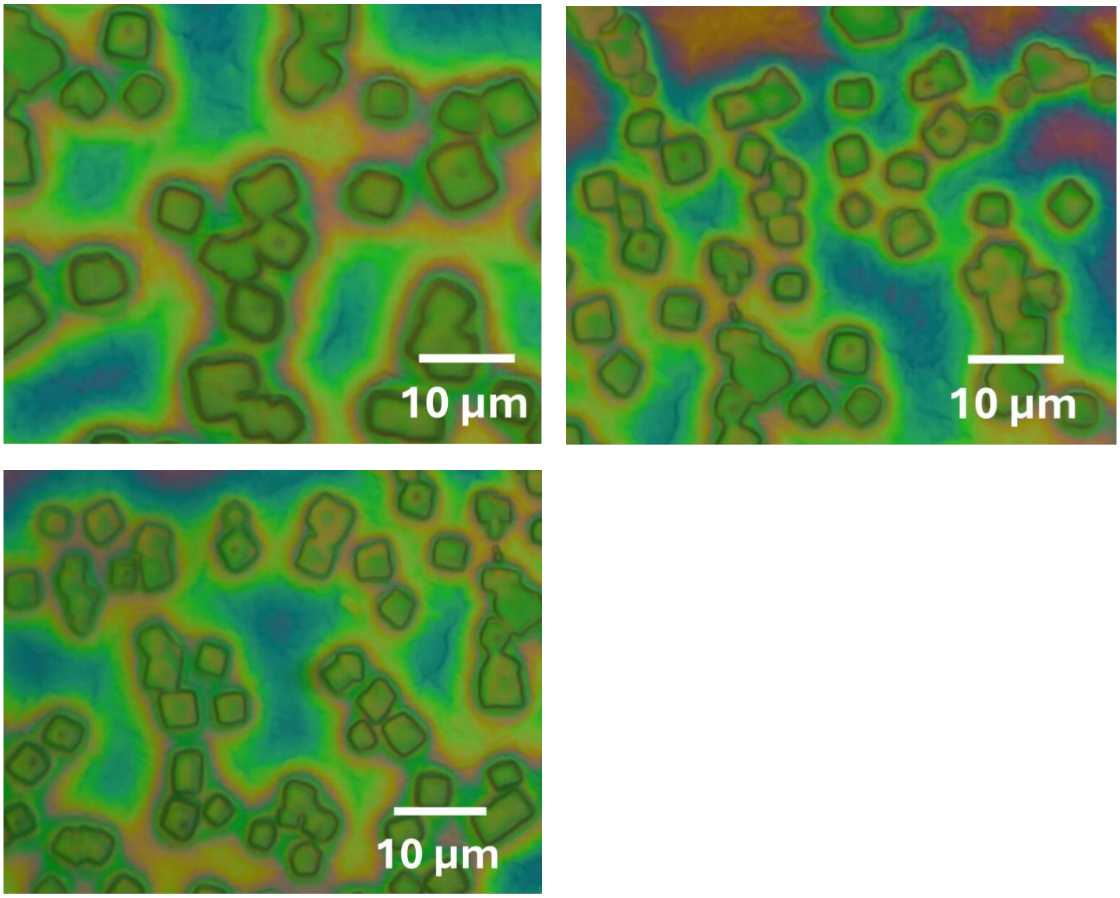

Figure S6. Optical images of Mn-doped (x = 0.17) NC superlattices at different locations.
